# Causal relationships between gut microbiota, C-reactive protein levels and colorectal cancer: A Mendelian randomization study

**DOI:** 10.1097/MD.0000000000049652

**Published:** 2026-07-03

**Authors:** Zhongxu Xing, Wei Gong, Yijun Xu, Yi Wu, Xiaoyan Xu, Songbing Qin, Yang Jiao, Lili Wang

**Affiliations:** a Department of Radiation Oncology, The First Affiliated Hospital of Soochow University, Suzhou, China; b State Key Laboratory of Radiation Medicine and Protection, School of Radiation Medicine and Protection, Collaborative Innovation Center of Radiological Medicine of Jiangsu Higher Education Institutions, Soochow University, Suzhou, China.

**Keywords:** C-reactive protein levels, Colorectal cancer, Gut microbiota, Mendelian randomization study

## Abstract

Gut microbiota have been associated with C-reactive protein (CRP) levels and colorectal cancer (CRC), but their causal relationships in humans remain unclear. We performed Mendelian randomization (MR) analyses to investigate causal relationships among gut microbiota, CRP, and CRC using genome-wide association studies (GWAS) summary data. The inverse variance weighted method was prespecified as the primary estimator, with complementary MR methods and sensitivity analyses used to assess robustness. Multiple-testing correction was applied across 209 gut microbial taxa. External validation and targeted replication were conducted using independent CRC GWAS datasets. An exploratory prerequisite-based analysis evaluated whether CRP might represent a potential inflammatory pathway linking CRC-associated gut microbial taxa to CRC. Five gut microbial taxa showed nominal associations with CRC. Genus *Eubacterium brachy group id.11296* (odds ratio [OR] = 1.13, 95% confidence intervals [CI] = 1.04–1.22, *P* = .002) and genus *Ruminococcaceae UCG004 id.11362* (OR = 1.15, 95% CI = 1.03–1.29, *P* = .016) were positively associated with CRC risk. Family *Enterobacteriaceae id.3469* (OR = 0.83, 95% CI = 0.69–1.00, *P* = .048), genus *Oscillibacter id.2063* (OR = 0.88, 95% CI = 0.77–1.00, *P* = .045), and order *Enterobacteriales id.3468* (OR = 0.83, 95% CI = 0.69–1.00, *P* = .048) showed inverse associations. However, none survived Bonferroni or Benjamini-Hochberg false discovery rate correction. Targeted replication provided partial support in BioBank Japan, with 3 taxa showing nominal replication, whereas no nominal replication was observed in FinnGen. For CRP, the weighted median method suggested a nominal inverse association with CRC risk, but this was not supported by the primary inverse variance weighting analysis or other complementary methods. The exploratory pathway analysis did not support CRP as a mediator linking the identified microbial taxa to CRC. This MR study identified 5 gut microbial taxa showing nominal associations with CRC risk, but these findings did not survive multiple-testing correction and should be interpreted as suggestive. Current evidence did not support a robust direct causal effect of CRP on CRC or a CRP-mediated microbiota-CRC pathway. Larger ancestry-matched GWAS datasets, strain-resolved microbiome analyses, and experimental studies are needed.

## 1. Introduction

Colorectal cancer (CRC) is a major global health challenge, maintaining its position as the second leading cause of cancer mortality worldwide.^[1]^ Notably, epidemiological trends reveal a concerning rise in early-onset CRC cases (<50 years), underscoring the critical importance of developing targeted prevention strategies.^[2]^ With approximately 50% of CRC cases estimated to be preventable through modifiable risk factors, identifying novel biomarkers and therapeutic targets emerges as a crucial public health priority.^[3]^ Hence, there is a pressing need for further research to identify biomarkers for early diagnosis and develop novel therapeutic targets for effective management of CRC.

The gut microbiota, residing in intimate proximity to the colorectal mucosa, comprises a complex ecosystem of microorganisms that maintain dynamic bidirectional communication with host cells. This intricate cross-talk regulates essential physiological processes, including immune modulation, metabolic homeostasis, and the integrity of the epithelial barrier.^[4]^ Advanced sequencing technologies have identified distinct microbial dysbiosis patterns in CRC patients, while experimental models have mechanistically linked specific bacterial species to carcinogenic processes.^[5,6]^ These findings underscore the dual potential of the gut microbiota as both a diagnostic biomarker and a therapeutic target, presenting opportunities for microbiome-based screening tools, prognostic indicators, and novel interventions ranging from cancer prevention to treatment optimization. Particularly relevant is the microbiota’s ability to modulate systemic inflammation through metabolic and immune pathways, potentially influencing circulating C-reactive protein (CRP) levels.^[7,8]^ Interestingly, serum concentrations of CRP have been associated with CRC risk and progression.^[9,10]^ Nevertheless, the relationship between the gut microbiota, CRP levels, and CRC development in humans remains predominantly unexplored, highlighting the imperative for a comprehensive investigation to elucidate potential clinical applications.

Genome-wide association studies (GWAS) have revolutionized our understanding of complex diseases by elucidating disease mechanisms and genetic risk factors.^[11]^ Mendelian randomization (MR) builds upon this foundation, employing genetic variants as instrumental variables (IVs) to establish causal relationships while minimizing confounding biases inherent in observational studies.^[12,13]^ In one-sample MR, both exposure and outcome data originate from the same individuals, whereas two-sample MR combines data from different sources to bolster statistical power.^[14,15]^ In our study, we conducted an extensive MR analysis utilizing comprehensive summary statistics to investigate the causal relationships between gut microbiota, CRP levels, and CRC. Additionally, we explored the potential of CRP levels to serve as an intermediary variable in the association between gut microbiota and CRC. Furthermore, we examined reverse causation by investigating whether genetic predisposition to CRC impacts gut microbiota and CRP levels. This multidimensional approach provides novel insights into the complex interplay between gut ecology, systemic inflammation, and colorectal carcinogenesis.

## 2. Methods

### 2.1. Research design

**Figure 1.**
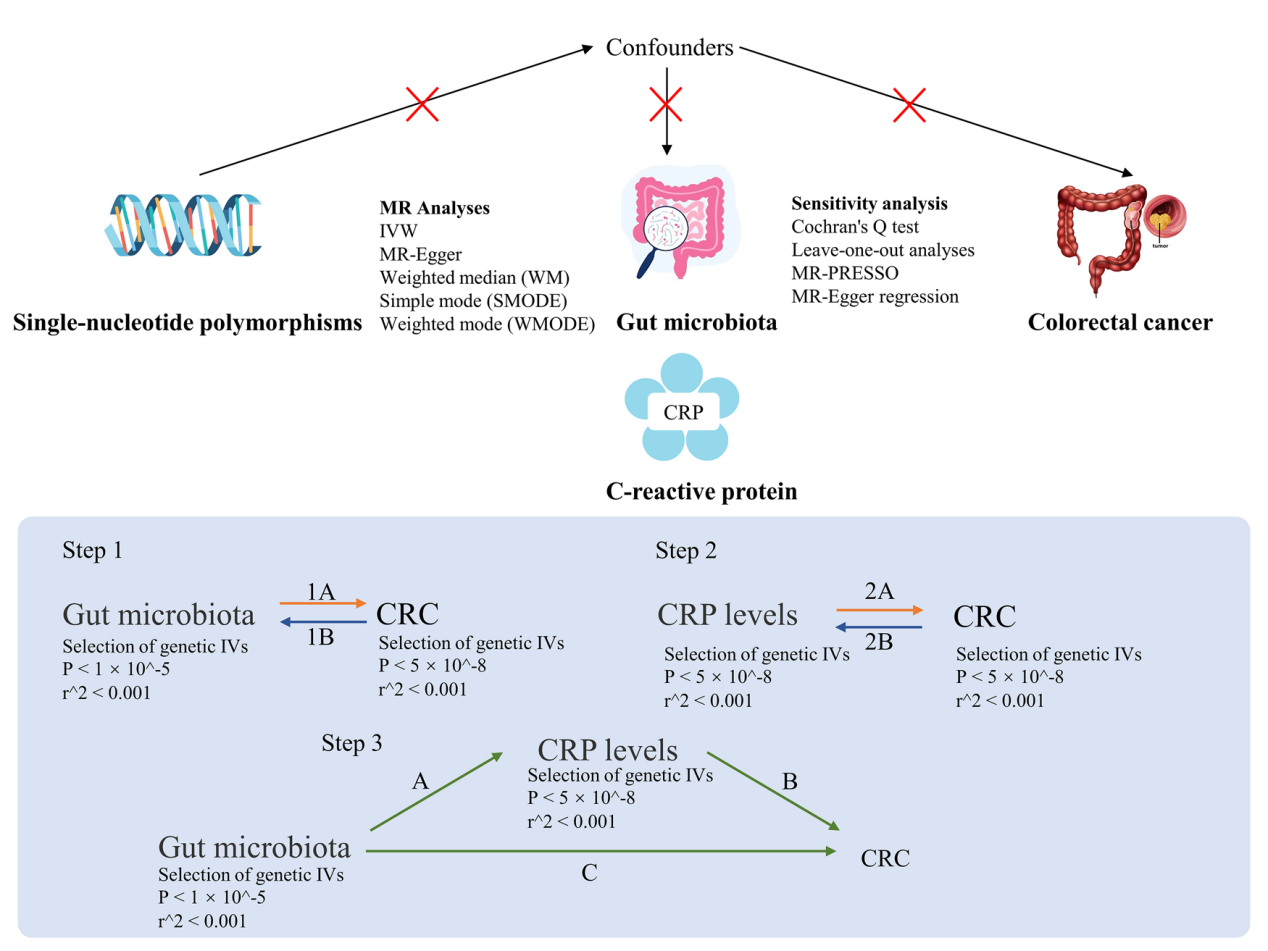
Schematic representation of Mendelian randomization analysis framework. In Step 1, causal dynamics between gut microbiota and CRC were investigated. Step 1A assessed the unidirectional effects of gut microbial communities on CRC risk, while Step 1B examined reciprocal causal relationships through bidirectional MR analysis. In Step 2, the association between CRP levels and CRC was studied. Step 2A investigated CRP’s causal effects on CRC development, and Step 2B analyzed potential reverse causation. Step 3 explored whether CRP could represent a potential systemic inflammatory pathway linking gut microbiota to CRC. Pathway C illustrates the hypothesized CRP-related pathway rather than a confirmed mediation mechanism. CRC = colorectal cancer, CRP = C-reactive protein, IVW = inverse variance weighting, MR = Mendelian randomization.

We performed a comprehensive MR study using European population GWAS data to investigate the causal relationships between gut microbiota, CRP levels, and CRC. The research comprises 3 sections, illustrated in Figure 1: causal assessment between 209 gut microbial taxa and CRC (Step 1A); (2) evaluation of CRP’s causal effects on CRC (Step 2A); and exploratory prerequisite-based assessment of the potential CRP-related inflammatory pathway (Step 3). Single-nucleotide polymorphisms (SNPs) were used as IVs to explore the causal relationship between exposure and outcome. MR relies on 3 fundamental assumptions: strong associations between IVs and exposure variables; IVs are entirely independent of confounding variables; and IVs do not have a direct effect on the outcome but only influence it through exposure.^[16]^

### 2.2. Data source

Microbiome genetic IVs were derived from the MiBioGen consortium’s GWAS meta-analysis (*N* = 18,340) comprising 211 microbial features across 9 phyla, 35 families, and 131 genera.^[17]^ After excluding microbial features without eligible instruments, 209 gut microbial taxa were included in the MR analysis. CRP genetic data originated from a GWAS of 575,531 individuals.^[18]^ The GWAS summary data of CRC were extracted from a study by Sakaue, accessible through the IEU Open GWAS database.^[19]^ Complete dataset specifications are provided in Supplementary Table S1, Supplemental Digital Content 7. For external validation and targeted replication, independent CRC GWAS datasets from UK Biobank, FinnGen, and BioBank Japan were used. The UK Biobank CRC GWAS dataset was used for external validation of the microbiota-CRC findings. FinnGen and BioBank Japan were used for targeted replication of the 5 gut microbial taxa highlighted in the primary analysis. Because this study was based exclusively on publicly available summary-level GWAS data, no additional ethical approval was required.

### 2.3. The selection of instrumental variables

For gut microbial taxa, SNPs associated with each taxon at a genome-wide suggestive significance threshold of *P* < 1 × 10^−5^ were selected as candidate instruments. This threshold was used because microbiome GWAS studies generally have relatively modest sample sizes, and applying the conventional genome-wide significance threshold of *P* < 5 × 10^−8^ would leave insufficient instruments for many taxa, thereby limiting the feasibility of multi-instrument MR and sensitivity analyses. For CRP, SNPs associated with circulating CRP levels at the conventional genome-wide significance threshold of *P* < 5 × 10^−8^ were selected as instruments. Following this, we implemented linkage disequilibrium (LD) clumping to ensure the independence of each IV associated with gut microbiota. The LD criteria used were *r*^2^ < 0.001 and distance > 10,000 kb.^[20]^ Aligning the effects of SNPs on exposure and outcome alleles was crucial to ensure that the selected SNPs were suitable for conducting the MR analysis effectively. Any palindromic SNPs, such as those with A/T or G/C alleles, were eliminated after matching with the outcome. Moreover, parameters like the explained variance (*R*^2^) and the *F*-statistic were computed to evaluate the association strength between identified IVs and exposure. IVs with F-statistic values below 10 were considered weak instruments in the study. Weak instruments can lead to biased or imprecise estimates in MR analyses, compromising the validity and robustness of causal inference.^[21]^ In this study, *R*^2^ was calculated using the formula *R^2^* = 2 ×* EAF* × (1–*EAF*) × *β*^2^/ (2 × *EAF* × (1–*EAF*) × *β*^2^ + 2 × *EAF* × (1–*EAF*) × *N* × standard error *[SE]*^2^). Here, *EAF* represents the effect allele frequency, *β* is the effect size, *N* is the sample size of the GWAS for the exposure of interest, and SE is the SE. The *F*-statistic was computed as *F* = *R*^2^ × (*N*–2)/ (1–*R*^2^).^[22]^ To further evaluate instrument-strength, we summarized SNP-level *F*-statistics and approximate variance explained for the 5 gut microbial taxa highlighted in the primary MR analysis. For each taxon, we reported the number of IVs, the minimum, median, mean, and maximum F-statistics, the range and median of approximate SNP-level *R*^2^ values, and the total approximate variance explained. Because the instruments were clumped using stringent LD criteria, the total approximate *R*^2^ for each exposure was calculated as the sum of the individual SNP-level *R*^2^ values. These results are presented in Supplementary Table S2–4, Supplemental Digital Content 8.

### 2.4. Mendelian randomization analysis

#### 2.4.1. Primary analysis

We employed the inverse variance weighting (IVW) method as the primary method for both gut microbiota-CRC and CRP-CRC analyses (Step 1A and Step 2A in Figure 1). For individuals with only one IV, the Wald ratio test was used for further examination. Complementary MR methods, including MR-Egger, weighted median (WM), simple mode, and weighted mode, were used as sensitivity estimators. The WM method was interpreted as a robustness analysis because it can provide valid estimates when at least 50% of the total instrumental-variable weight is derived from valid instruments. However, it was not considered the primary estimator. Therefore, causal interpretation was primarily based on the IVW estimate, multiple-testing-corrected results, and consistency across complementary MR methods. Results are reported as odds ratios (ORs) with 95% confidence intervals (CIs). For the gut microbiota-CRC analysis, 209 microbial taxa were evaluated. Therefore, multiple-testing correction was performed based on the primary IVW *P*-values. The Bonferroni-corrected significance threshold was defined as *P* < .05/209 = 2.39 × 10^−4^. Benjamini-Hochberg false discovery rate (FDR) correction was also applied, with FDR-adjusted *q* < 0.05 considered statistically significant. Associations with unadjusted *P* < .05 but not meeting Bonferroni or FDR correction were considered nominal or suggestive findings rather than definitive causal evidence.

#### 2.4.2. Exploratory CRP-related pathway analysis

After identifying gut microbial taxa showing putative associations with CRC, we conducted an exploratory prerequisite-based analysis to evaluate whether CRP might represent a potential systemic inflammatory pathway linking gut microbiota to CRC. First, we tested whether the CRC-associated gut microbial taxa were causally associated with circulating CRP levels. A statistically significant microbiota-to-CRP association was considered a prerequisite for further interpretation of a CRP-mediated pathway.

If this prerequisite had been met, the potential mediated effect would have been evaluated by combining the microbiota-to-CRP and CRP-to-CRC estimates. If no significant microbiota-to-CRP association was observed, the analysis was interpreted as not supporting the prerequisite for CRP-mediated mediation under the current MR framework. In that case, no mediation proportion was estimated or interpreted.

#### 2.4.3. Bi‑directional causality analysis

Reverse causation was assessed by designating CRC as exposure and significant gut microbiota/CRP features as outcomes (Fig. 1). IVs were chosen based on their significant associations with CRC (*P* < 5 × 10^−8^) to facilitate this analysis. The same LD clumping, harmonization, and weak-instrument exclusion procedures were applied. If no eligible SNPs remained after harmonization, reliable reverse MR estimates were not generated.

### 2.5. Sensitivity analysis

We conducted thorough sensitivity analyses to ensure the robustness of our findings. First, we employed Cochran’s *Q* test to assess heterogeneity for each SNP. Second, scatter plots were generated to visually represent the associations between SNPs and exposure, as well as SNPs and outcomes, thereby illustrating the MR results.^[23]^ Third, leave-one-out analyses were performed to evaluate the impact of individual SNPs on the overall results; in this process, each SNP was systematically excluded, and an IVW method was applied to the remaining SNPs for assessment.^[24]^ Finally, we utilized MR-PRESSO and MR-Egger regression techniques to identify and manage horizontal pleiotropy effects, further ensuring the robustness of our study. Noteworthy outliers identified by MR-PRESSO were addressed by removing them to correct for horizontal pleiotropy.^[25]^

### 2.6. External validation and targeted replication

To further evaluate the robustness of the microbiota-CRC findings, external validation was performed using an independent CRC GWAS dataset from the UK Biobank. MR analyses were conducted using the same gut microbial instruments as in the primary analysis, with CRC status in the UK Biobank as the outcome. Sensitivity analyses, including MR-Egger regression, heterogeneity testing, and leave-one-out analysis, were performed where applicable.

In addition, targeted external replication was conducted for the 5 gut microbial taxa highlighted in the primary analysis using independent CRC GWAS datasets from FinnGen and BioBank Japan. The same exposure instruments were used, and outcome associations were extracted from locally downloaded GWAS-VCF files. Harmonization and MR analyses were performed using the TwoSampleMR package. Nominal replication was defined as *P* < .05 with a direction of effect consistent with the discovery analysis. Because 5 taxa were evaluated in the targeted replication analysis, Benjamini-Hochberg FDR and Bonferroni corrections were applied across the 5 targeted taxa.

### 2.7. Statistical software

All MR analyses were performed using R software version 4.2.1. Genetic instruments were extracted and harmonized using the TwoSampleMR package version 0.6.29. Horizontal pleiotropy was assessed using the MRPRESSO package. Multiple-testing correction was performed using the *P*.adjust function in R with the Bonferroni and Benjamini-Hochberg FDR methods. Scatter plots, forest plots, and leave-one-out plots were generated using TwoSampleMR and ggplot2.

## 3. Results

### 3.1. Instrumental variable selection

Genome-wide association analysis initially identified 190, 410, 1416, 230, and 108 SNPs associated with 209 gut microbiota taxa at class, family, genus, order, and phylum taxonomic levels, respectively, using a genome-wide suggestive significance threshold of *P <* 1 × 10^−5^. Two gut microbial features were excluded from subsequent analysis because no eligible SNPs were available. After rigorous quality-control procedures, 2354 independent SNPs met the predefined selection criteria and were retained as IVs for MR analysis of 209 gut microbiota exposures (refer to Supplementary Table S5, Supplemental Digital Content 11). Additionally, 264 genome-wide significant SNPs (*P* < 5 × 10^−8^) associated with CRP levels were identified as IVs for MR analysis (refer to Supplementary Table S6, Supplemental Digital Content 12).

Instrument-strength summaries for the 5 gut microbial taxa highlighted in the primary MR analysis are provided in Supplementary Table S7, Supplemental Digital Content 13. These taxa were instrumented by 6 to 14 SNPs. All SNP-level *F*-statistics were above the conventional threshold of 10, with values ranging from 19.06 to 29.13 across all instruments. The median F-statistics for individual taxa ranged from 19.69 for *Enterobacteriaceae*/*Enterobacteriales* to 21.88 for *Oscillibacter*. The total approximate variance explained by the instruments was modest, ranging from 0.00681 for *Enterobacteriaceae*/*Enterobacteriales* to 0.01693 for *Oscillibacter*. These results indicate acceptable instrument-strength according to conventional *F*-statistic criteria, while also highlighting the limited variance explained by microbiome GWAS instruments.

### 3.2. Primary MR analysis of gut microbiota and CRP levels on CRC

**Figure 2.**
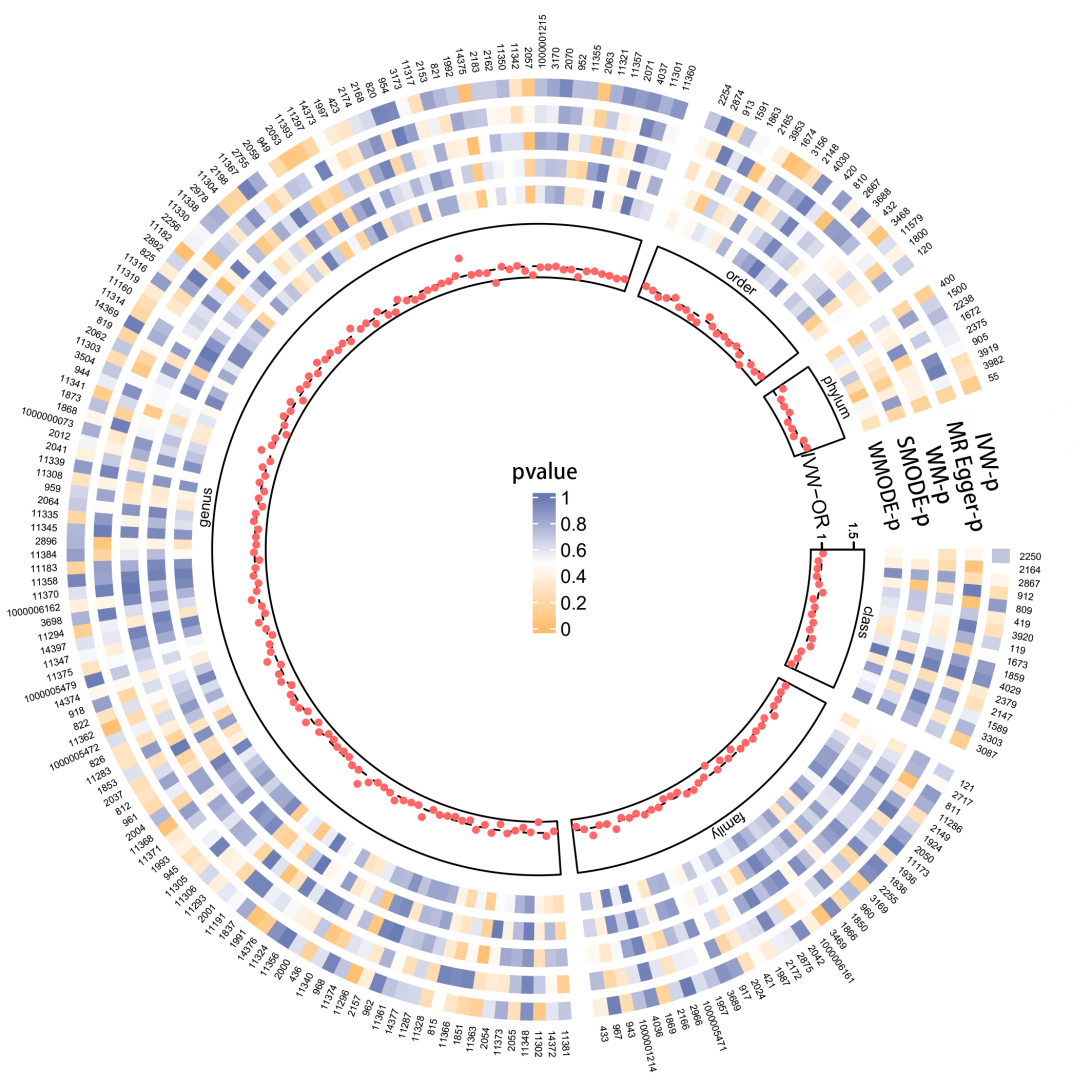
Mendelian randomization analysis of gut microbiota’s causal effects on CRC. The radial plot demonstrates results from 5 MR methods, with red dots representing SNP-specific effect estimates on CRC risk calculated through the IVW method. Different sections of the graph correspond to various levels of gut microbiota, such as order, phylum, genus, class, and family. The dashed reference line indicates a null effect (OR = 1). Peripheral concentric circles display *P*-values from IVW, MR-Egger, WM, SMODE, and WMODE methods. The outermost ring lists microbial IDs cross-referenced with full taxonomic nomenclature in Supplementary Table S16, Supplemental Digital Content 22. CRC = colorectal cancer, IVW = inverse variance weighting, OR = odds ratio, SNP = single-nucleotide polymorphisms, SMODE = simple mode, WM = weighted median, WMODE = weighted mode.

**Figure 3.**
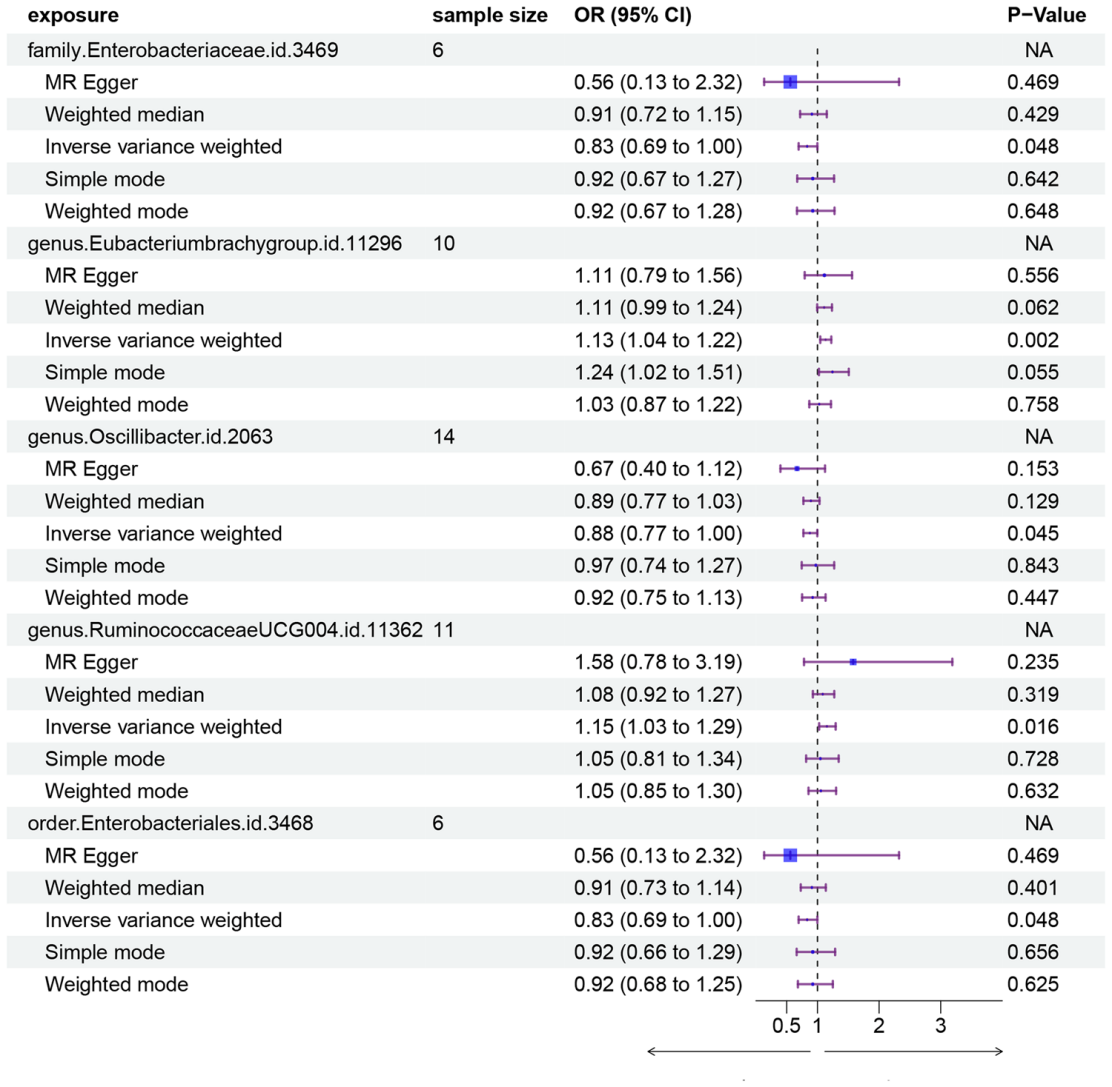
Forest plot of Mendelian randomization estimates for gut microbiota–colorectal cancer associations. Odds ratios and 95% confidence intervals were calculated from the IVW method as the primary MR analysis. For taxa with only one available genetic instrument, the Wald ratio method was used. Effect estimates are shown as ORs for colorectal cancer per genetically predicted increase in microbial abundance. The 95% confidence intervals were calculated as exp (β ± 1.96 × SE). Two-sided *P*-values from the corresponding MR method are displayed alongside the estimates. The vertical dashed line indicates the null effect, OR = 1. The 5 taxa shown in Figure 3. Were further evaluated in targeted external replication analyses using FinnGen and BioBank Japan colorectal cancer GWAS datasets. CI = confidence intervals, IVW = inverse variance weighting, MR = Mendelian randomization, OR = odds ratio.

Using the IVW method as the primary estimator, we first screened 209 gut microbial taxa for their associations with CRC risk. An overview of the MR estimates is presented in Figure 2. Five taxa showed nominal associations with CRC at the unadjusted *P* < .05 level and demonstrated directional consistency across complementary MR methods. However, after correction for multiple-testing across 209 taxa, none of these associations reached the Bonferroni-corrected significance threshold of *P* < 2.39 × 10^−4^ or the Benjamini-Hochberg FDR threshold of *q* < 0.05. Therefore, these findings should be interpreted as suggestive rather than definitive causal evidence (refer to Figure 3). Specifically, 2 microbial genera exhibited nominal positive associations with CRC risk: genus *Eubacteriumbrachygroup id.11296* (OR = 1.13, 95% CI = 1.04–1.22, *P* = .002) and genus *RuminococcaceaeUCG004 id.11362* (OR = 1.15, 95% CI = 1.03–1.29, *P* = .016). Conversely, 3 taxa showed nominal inverse associations with CRC risk: family *Enterobacteriaceae id.3469* (OR = 0.83, 95% CI = 0.69–1.00, *P* = .048), genus *Oscillibacter id.2063* (OR = 0.88, 95% CI = 0.77–1.00, *P* = .045), and order *Enterobacteriales id.3468* (OR = 0.83, 95% CI = 0.69–1.00, *P* = .048).

Because these associations did not survive multiple-testing correction, they were interpreted as suggestive rather than conclusive causal findings. The inverse estimates for family *Enterobacteriaceae* and order *Enterobacteriales* were therefore reported as nominal inverse associations with CRC risk, rather than definitive evidence of a protective biological effect. Given the hierarchical relationship between these 2 taxa, these results may reflect an overlapping taxonomic signal.

**Figure 4.**
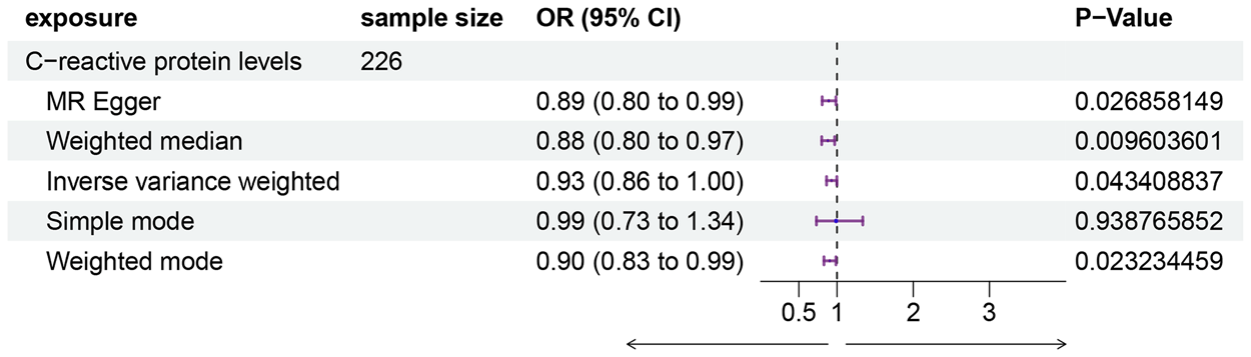
Forest plot of Mendelian randomization estimates for CRP levels and colorectal cancer risk. MR estimates were generated using IVW, MR-Egger, weighted median, simple mode, and weighted mode methods. Because heterogeneity was detected in the CRP-CRC analysis, complementary estimators were used to assess the robustness of the IVW result. The weighted median estimate was interpreted as a sensitivity finding rather than as primary evidence. Odds ratios and 95% confidence intervals were calculated as exp(β) and exp(β ± 1.96 × SE), respectively. Two-sided *P*-values are shown for each method. The vertical dashed line indicates the null effect, OR = 1. Although the weighted median method suggested an inverse association, this result was interpreted as a sensitivity finding because it was not consistently supported by the primary IVW analysis and other complementary MR methods. CRP = C-reactive protein, IVW = inverse variance weighting, MR = Mendelian randomization, OR = odds ratio.

In the CRP-CRC analysis shown in Figure 4, the WM estimator yielded a nominal inverse association between genetically predicted CRP levels and CRC risk (OR = 0.88, 95% CI = 0.80–0.97, *P* < .05). However, this association was not corroborated by the prespecified primary IVW analysis or by other complementary MR estimators, including MR-Egger. Given the inconsistency across MR methods, this finding was interpreted as an exploratory sensitivity result rather than robust evidence for a direct causal effect of CRP on CRC risk.

### 3.3. Sensitivity analyses

**Figure 5.**
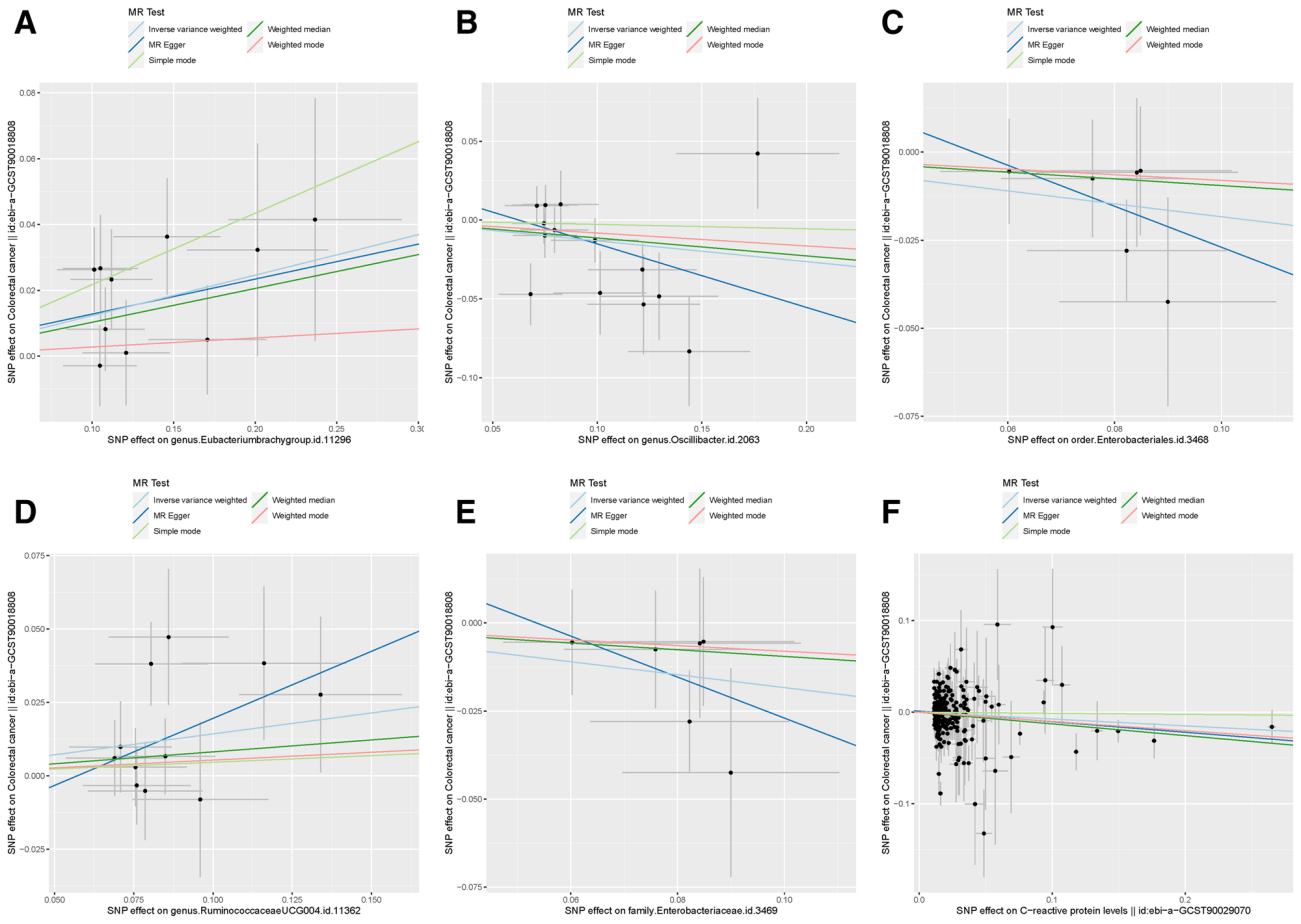
Scatter plots of Mendelian randomization analyses in the discovery dataset. Panels (A–E) show SNP-specific associations between genetically predicted gut microbial taxa and colorectal cancer risk. Panel F shows the association between genetically predicted CRP levels and colorectal cancer risk. The x-axis represents the SNP effect on the exposure, and the y-axis represents the SNP effect on the outcome. Each point represents 1 instrumental variable. The fitted lines correspond to estimates from complementary MR methods, including IVW, MR-Egger, weighted median, simple mode, and weighted mode. These scatter plots were generated from the discovery dataset only. CI = confidence intervals, CRP = C-reactive protein, IVW = inverse variance weighting, MR = Mendelian randomization, OR = odds ratio, SNP = single-nucleotide polymorphisms.

For the 5 gut microbiota-CRC associations highlighted in the primary MR analysis, multiple sensitivity analyses were conducted to evaluate the robustness of the findings. The MR Egger test confirmed the absence of horizontal pleiotropy in the gut microbiota and CRC MR analysis (all *P* > .05, Supplementary Table S8, Supplemental Digital Content 14). Cochran’s *Q* tests did not indicate significant heterogeneity among the IVs (all *P* > .05), suggesting that the SNP-specific estimates were generally consistent. Leave-one-out analyses further showed that no single SNP drove the observed associations (Supplementary Figure S1–5, Supplemental Digital Content 1). Scatter plots illustrating the SNP-specific effects for the 5 highlighted gut microbial taxa did not reveal obvious outliers among the IVs (Fig. 5). Together, these sensitivity analyses provided supportive evidence for the stability of the suggestive microbiota-CRC associations, although the results should still be interpreted cautiously because they did not pass multiple-testing correction.

In the MR analysis of CRP levels and CRC, Cochran’s *Q* test suggested heterogeneity among the CRP-associated IVs (Supplementary Table S9, Supplemental Digital Content 15). MR-Egger regression did not detect evidence of directional pleiotropic bias (*P* > .05; Supplementary Table S10, Supplemental Digital Content 16), and the leave-one-out analysis indicated that the overall conclusion was not driven by any single SNP (Supplementary Figure S6, Supplemental Digital Content 6). However, because the CRP-CRC association was not consistently reproduced across the primary IVW analysis and complementary MR methods, the sensitivity analyses did not support a robust direct causal relationship between genetically predicted CRP levels and CRC risk.

### 3.4. Bi‑directional MR analysis

We next performed bidirectional MR analysis to evaluate whether genetic liability to CRC could influence gut microbiota composition or CRP levels. For the CRC-to-gut microbiota analyses, no eligible SNPs remained after harmonization under the predefined instrumental-variable selection criteria. Therefore, reliable reverse MR estimates could not be obtained for CRC effects on the 5 highlighted gut microbial taxa. For the CRC-to-CRP analysis, the MR results did not provide evidence supporting a significant reverse causal effect of CRC liability on CRP levels (Supplementary Table S11, Supplemental Digital Content 17). When harmonizing CRC-associated gut microbiota data, no SNPs met the stringent criteria for valid IVs. Similarly, MR analyses demonstrated no evidence of reverse causation between CRP levels and CRC risk.

### 3.5. Exploratory assessment of the CRP-related pathway

We performed an exploratory, prerequisite-based assessment to determine whether CRP could represent an inflammatory pathway linking the 5 CRC-associated microbial taxa to CRC. In this framework, a significant microbiota-to-CRP association was required before a CRP-mediated pathway could be formally interpreted. However, none of the 5 CRC-associated microbial taxa showed a statistically significant causal association with CRP levels. Therefore, the prerequisite for a formal CRP-mediated pathway was not met, and no mediation proportion was interpreted.

These findings do not provide evidence supporting CRP as a mediator of the observed microbiota-CRC associations in the current GWAS-based MR framework. However, this result should be interpreted as an exploratory assessment of one systemic inflammatory biomarker rather than definitive evidence excluding inflammatory mediation. Other, more specific inflammatory mediators, such as cytokine-related pathways, may still be involved and warrant further investigation using well-powered and ancestry-matched GWAS datasets.

### 3.6. External validation

Independent validation was first performed using the UK Biobank CRC GWAS dataset (*N* = 5657). Among the 5 gut microbial taxa highlighted in the discovery analysis, genus *Oscillibacter id.2063* showed an inverse association with CRC risk consistent with the primary MR result (Supplementary Table S12, Supplemental Digital Content 18). Sensitivity analyses did not detect evidence of horizontal pleiotropy or significant heterogeneity for this validation result, supporting its robustness within the UK Biobank dataset (Supplementary Table S13, Supplemental Digital Content 19). However, because only one of the 5 highlighted taxa was supported in this validation analysis, the UK Biobank results should be interpreted as partial validation rather than confirmation of all primary findings.

We further performed targeted external replication for the 5 taxa highlighted in Figure 3 using CRC GWAS datasets from FinnGen and BioBank Japan. All 5 taxa were available for replication in both datasets. In BioBank Japan, all 5 taxa showed effect estimates in the same direction as the discovery analysis, and 3 reached nominal significance: genus *Ruminococcaceae UCG004 id.11362* (OR = 1.20, 95% CI = 1.02–1.42, *P* = .031), family *Enterobacteriaceae id.3469* (OR = 0.755, 95% CI = 0.576–0.991, *P* = .043), and order *Enterobacteriales id.3468* (OR = 0.755, 95% CI = 0.576–0.991, *P* = .043) (Supplementary Table S14, Supplemental Digital Content 20). However, none of these associations survived targeted FDR or Bonferroni correction.

In FinnGen, all 5 taxa were also available for analysis, but only 2 showed directionally consistent estimates, and none reached nominal significance (Supplementary Table S15, Supplemental Digital Content 21). Overall, the targeted replication analyses provided partial support for the Figure 3 findings, particularly in BioBank Japan, but did not confirm the associations after multiple-testing correction or consistently across both replication datasets. These findings further support a cautious interpretation of the primary MR results and highlight the need for additional validation in larger and ancestry-matched datasets.

## 4. Discussion

The gut microbiota represents a complex and dynamic microbial ecosystem that plays an important role in maintaining intestinal homeostasis, host metabolism, immune regulation, and mucosal barrier integrity.^[26]^ Through continuous interactions with intestinal epithelial cells and immune components, gut microbial communities may influence local inflammatory responses, metabolic signaling, and carcinogenesis-related biological processes.^[4]^ Chronic inflammation has also been proposed as a potential interface between microbial dysbiosis and colorectal carcinogenesis, and systemic inflammatory biomarkers such as CRP have been associated with cancer risk in observational studies.^[27]^ However, observational associations may be affected by confounding, reverse causation, and disease-related inflammatory responses. Therefore, MR provides a useful genetic epidemiological framework to evaluate whether gut microbiota and CRP levels may have potential causal relevance to CRC.

In this investigation, we utilized MR analysis to examine possible causal relationships between 209 gut microbial taxa and CRC risk. We identified 5 gut microbial taxa showing nominal associations with CRC risk. Two genus-level taxa, *Eubacterium brachy group id.11296* and *Ruminococcaceae UCG004 id.11362*, were positively associated with CRC risk, whereas family *Enterobacteriaceae id.3469*, genus *Oscillibacter id.2063*, and order *Enterobacteriales id.3468* showed inverse associations with CRC risk. Importantly, none of these associations survived Bonferroni or Benjamini-Hochberg FDR correction across 209 microbial taxa. Therefore, these findings should be interpreted as suggestive and hypothesis-generating rather than definitive causal evidence. Nevertheless, the absence of clear directional pleiotropy, the generally stable leave-one-out results, and the consistency of some findings in external datasets provide preliminary support for further investigation of these candidate taxa. The 2 taxa positively associated with CRC risk may have biological relevance, although interpretation should remain cautious. The genus *Eubacterium* is a heterogeneous microbial group involved in several physiological processes, including immune regulation, gut barrier function, metabolic homeostasis, and inflammatory modulation.^[28]^ Furthermore, *Eubacterium* species have been implicated in cancer initiation through inflammation promotion.^[29]^ Therefore, the observed positive association for *Eubacterium brachy group* should not be generalized to all members of the genus. Instead, it may reflect a more specific host–microbiota ecological pattern that requires species- or strain-level validation. The family *Ruminococcaceae* has been significantly associated with inflammatory bowel disease.^[30]^ Previous research has demonstrated a distinct elevation in the abundance of *Ruminococcaceae* in the mucosa of CRC patients compared to normal controls.^[31]^ The positive association observed for *Ruminococcaceae UCG004* may therefore be biologically plausible, but the broad taxonomic resolution of the available microbiome GWAS data limits mechanistic interpretation.

Among the taxa showing inverse associations, *Oscillibacter* may be of particular interest. *Oscillibacter* has been linked to tumor growth suppression in preclinical studies when mice were fed a probiotic mixture.^[32,33]^ Analysis of gut microbiota profiles revealed significant enrichment of *Oscillibacter* and other species in treated mice, suggesting a potential role in tumor suppression. An increase in *Oscillibacter* was associated with a reduction in tumor-infiltrating Th17 cells.^[32]^ In contrast, patients with gastric cancer and gastrointestinal stromal tumors observed lower *Oscillibacter* abundance compared to healthy controls.^[34]^ *Oscillibacter* is known to produce anti-inflammatorymetabolites and contribute to maintaining gut barrier integrity in mice.^[35]^ These findings provide a possible biological basis for the inverse association observed in our MR analysis. However, because the present result did not survive multiple-testing correction and was based on genetically proxied relative abundance rather than direct microbial measurement, it should be interpreted as a suggestive inverse association rather than evidence that *Oscillibacter* is a confirmed protective factor.

The role of *Enterobacteriaceae* in malignancies has garnered increased attention, with *Enterobacteria* observed to decrease in patients with CRC.^[36]^ On the contrary, *Enterobacteria* may serve as significant cofactors in dysplasia formation through various mechanisms, such as triggering or amplifying inflammatory responses or releasing harmful agents that cause DNA damage or genetic instability.^[37]^ For example, pks-positive *E coli* can produce colibactin and has been linked to CRC-related mutational processes. Therefore, our findings should not be interpreted as evidence that *Enterobacteriaceae* or *Enterobacteriales* are biologically protective against CRC. Several factors may explain this apparent discrepancy. First, *Enterobacteriaceae* and *Enterobacteriales* are broad taxonomic categories and do not distinguish between commensal and pathogenic strains. Second, *Enterobacteriaceae* is nested within the broader order *Enterobacteriales*, and the similar effect estimates observed for these 2 taxa may reflect overlapping taxonomic signals rather than 2 independent microbial effects. Third, the MR framework estimates associations between host genetic variants linked to relative microbial abundance and disease risk; it does not capture microbial functional activity, virulence factors, pks/colibactin status, biofilm formation, mucosal localization, or inflammatory potential. Thus, genetically predicted lower abundance of a broad taxonomic group cannot be directly equated with reduced pathogenic activity or a clinically protective effect.

External validation and targeted replication provided mixed but informative evidence. In the UK Biobank validation analysis, *Oscillibacter* showed an inverse association with CRC risk consistent with the discovery analysis, and sensitivity analyses did not indicate substantial pleiotropy or heterogeneity. This result provides partial support for the primary findings but does not confirm all 5 candidate taxa. In BioBank Japan, all 5 taxa showed effect estimates in the same direction as the discovery analysis, and 3 reached nominal significance. However, none survived the targeted FDR or Bonferroni correction. In FinnGen, only 2 taxa showed directionally consistent estimates, and none reached nominal significance. These differences may reflect variation in ancestry, phenotype definition, statistical power, allele frequencies, LD structure, or the number of available instruments after harmonization. In particular, the BBJ analysis should be viewed as a trans-ancestry sensitivity analysis rather than a direct same-ancestry replication. Overall, the external analyses provide partial support for the candidate microbiota-CRC associations, but they also reinforce the need for validation in larger, ancestry-matched datasets.

The CRP-related findings should also be interpreted cautiously. Although the WM estimator suggested a nominal inverse association between genetically predicted CRP levels and CRC risk, this association was not corroborated by the prespecified primary IVW analysis or by other complementary MR methods, including MR-Egger. Because IVW was defined as the primary estimator and WM was used as a sensitivity estimator, the WM finding alone was not considered sufficient evidence for a robust direct causal effect of CRP on CRC risk. This method-specific result may reflect estimator-specific assumptions, instrument heterogeneity, or residual pleiotropy. Therefore, CRP should not be interpreted as a confirmed causal protective factor for CRC based on the current MR evidence.

These CRP findings are relevant to the interpretation of the mediation analysis. We hypothesized that CRP might represent a systemic inflammatory pathway linking gut microbiota to CRC. However, none of the 5 CRC-associated microbial taxa showed a statistically significant causal association with CRP levels. Therefore, the prerequisite for a formal CRP-mediated pathway was not met, and no mediation proportion was estimated or interpreted. The present findings do not support CRP as a mediator linking the identified gut microbial taxa to CRC in the current GWAS-based MR framework. However, this should be interpreted as a lack of evidence for CRP-mediated mediation rather than evidence excluding inflammatory mechanisms altogether. CRP is a broad systemic inflammatory biomarker and may not adequately capture local mucosal inflammation or more specific cytokine-mediated pathways, such as IL-6, TNF-α, or other immune signaling mechanisms. Future studies incorporating well-powered GWAS data for more specific inflammatory biomarkers may help clarify whether alternative inflammatory pathways mediate microbiota-CRC associations.

Bidirectional MR analyses did not provide clear evidence of reverse causality, but this conclusion should be stated with caution. For the CRC-to-gut microbiota analyses, no eligible SNPs remained after harmonization under the predefined instrumental-variable selection criteria; therefore, reliable reverse MR estimates could not be obtained for CRC effects on the highlighted gut microbial taxa. For the CRC-to-CRP analysis, the MR results did not support a significant reverse causal effect of CRC liability on CRP levels. Thus, while the available evidence did not indicate a clear reverse causal relationship, the reverse analysis for gut microbiota was limited by the lack of eligible instruments.

This study has several strengths. First, we used large GWAS summary datasets to systematically evaluate the relationships among gut microbial taxa, CRP levels, and CRC risk. Second, we implemented a comprehensive MR framework, including primary MR analysis, complementary MR estimators, bidirectional analysis, and multiple sensitivity analyses to assess heterogeneity, horizontal pleiotropy, and the influence of individual SNPs. Third, external validation and targeted replication were performed using independent CRC GWAS datasets, including UK Biobank, FinnGen, and BioBank Japan. Fourth, we explicitly considered multiple-testing correction and distinguished nominal findings from corrected statistical significance, thereby reducing the risk of overinterpretation.

Several limitations should also be acknowledged. First, because microbiome GWAS studies often have relatively limited sample sizes, a genome-wide suggestive threshold was used to select instruments for gut microbial taxa. Although all retained instruments had F-statistics above the conventional threshold of 10, the variance explained by the instruments was modest, and residual weak-instrument bias or false-positive findings cannot be fully excluded. Second, none of the 5 microbiota-CRC associations survived Bonferroni or FDR correction across 209 taxa, and targeted replication did not consistently confirm the findings after multiple-testing correction. Therefore, the microbiota-related results should be interpreted as exploratory and hypothesis-generating. Third, the microbial exposures were defined by genetically proxied relative abundance of broad taxonomic groups. These proxies do not distinguish between species, strains, commensal and pathogenic members, virulence factors, toxin production, mucosal colonization, or microbial functional activity. This limitation is particularly important for interpreting the inverse associations observed for *Enterobacteriaceae* and *Enterobacteriales*. Fourth, because the analyses were based on summary-level GWAS data, we could not perform individual-level stratified analyses by diet, medication use, antibiotic exposure, tumor site, tumor stage, or measured CRP concentrations. Fifth, the primary GWAS datasets were mainly based on individuals of European ancestry. Although BBJ provided a trans-ancestry sensitivity analysis, differences in allele frequencies, LD patterns, microbiome composition, dietary habits, lifestyle factors, and environmental exposures may limit generalizability to non-European populations. Finally, CRP was the only inflammatory biomarker assessed in the mediation framework. Other inflammatory mediators, including IL-6, TNF-α, and additional cytokine-related pathways, were not systematically evaluated and may provide further mechanistic insight.

In summary, this MR study identified several gut microbial taxa showing nominal associations with CRC risk and provided preliminary evidence that some of these associations may be directionally reproducible in external datasets. However, the findings did not survive multiple-testing correction and were not consistently replicated across all validation datasets. The CRP-related analyses did not support CRP as a robust causal factor for CRC or as a mediator linking gut microbiota to CRC. Future studies integrating larger ancestry-diverse microbiome GWAS datasets, ancestry-matched CRC GWAS datasets, strain-resolved metagenomics, metatranscriptomics, metabolomics, cytokine profiling, and experimental models are needed to validate these candidate microbial taxa and clarify the biological pathways underlying microbiota-related colorectal carcinogenesis.

## 5. Conclusion

In this study, we conducted a comprehensive MR analysis to evaluate the potential causal relationships among gut microbiota, CRP levels, and CRC. We identified 5 gut microbial taxa showing nominal associations with CRC risk: 2 positively associated taxa, genus *Eubacterium brachy group id.11296* and genus *Ruminococcaceae UCG004 id.11362*, and 3 inversely associated taxa, family *Enterobacteriaceae id.3469*, genus *Oscillibacter id.2063*, and order *Enterobacteriales id.3468*. However, none of these associations survived Bonferroni or Benjamini-Hochberg FDR correction across 209 taxa. Therefore, these findings should be regarded as suggestive and hypothesis-generating rather than definitive causal evidence. External validation and targeted replication provided partial support, particularly for *Oscillibacter* in the UK Biobank and for directionally consistent estimates in BioBank Japan, but the associations were not consistently replicated after multiple-testing correction. The CRP-related analyses did not provide robust evidence that CRP has a direct causal effect on CRC or mediates the relationship between gut microbiota and CRC. Overall, our findings suggest that specific gut microbial taxa may be relevant to CRC development, but larger ancestry-matched GWAS datasets, strain-resolved multi-omics studies, and experimental validation are needed to confirm these associations and clarify the underlying biological mechanisms.

## Acknowledgments

This work was supported by the National Natural Science Foundation of China (82073476), the National Key R&D Program of China (2022YFC2503700, 2022YFC2503703), Jiangsu Provincial Medical Key Discipline (ZDXK202235), Innovation Research Project of Medical and Industrial Cooperation in Suzhou (SLJ2021005), Project of the State Key Laboratory of Radiation Medicine and Protection, Soochow University (GZC00402, GZN1202101) and the Priority Academic Program Development (PAPD) of Jiangsu Higher Education Institutions.

## Author contributions

**Formal analysis:** Zhongxu Xing, Wei Gong.

**Data curation:** Yijun Xu.

**Investigation:** Yijun Xu, Yi Wu.

**Supervision:** Yang Jiao, Lili Wang.

**Conceptualization:** Lili Wang.

**Writing – original draft:** Zhongxu Xing, Wei Gong.

**Writing – review & editing:** Xiaoyan Xu, Songbing Qin, Yang Jiao, Lili Wang.












































