# Supplementary figures and images for: Causal relationships between gut microbiota, C-reactive protein levels and colorectal cancer: A Mendelian randomization study
Source: Medicine (Baltimore). 2026 Jul 3;105(27):e49652. doi: 10.1097/MD.0000000000049652 (PMC13337044; doi:10.1097/MD.0000000000049652)

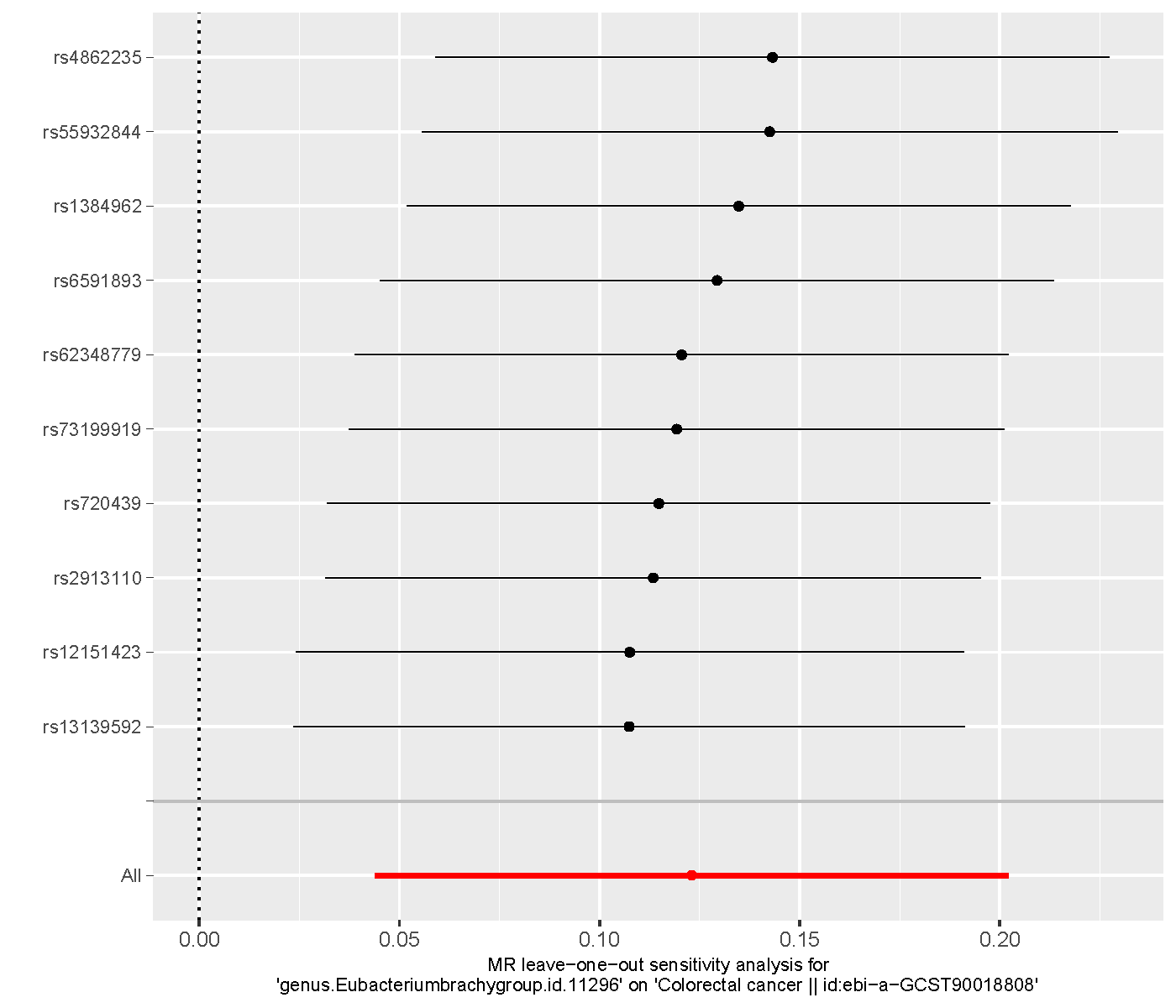

Supplement: Supplementary file 1 [file medi-105-e49652-s001.tiff]

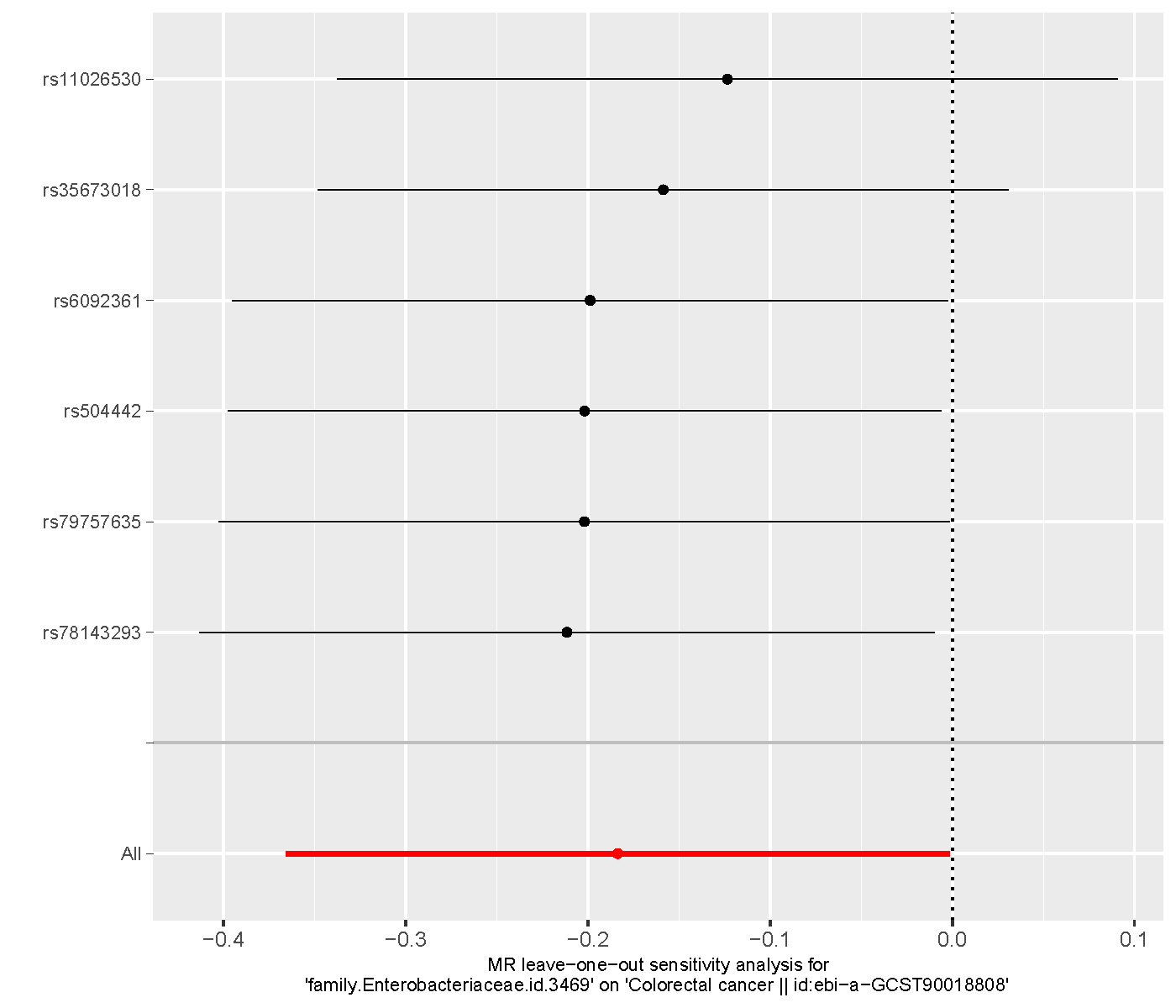

Supplement: Supplementary file 2 [file medi-105-e49652-s002.tiff]

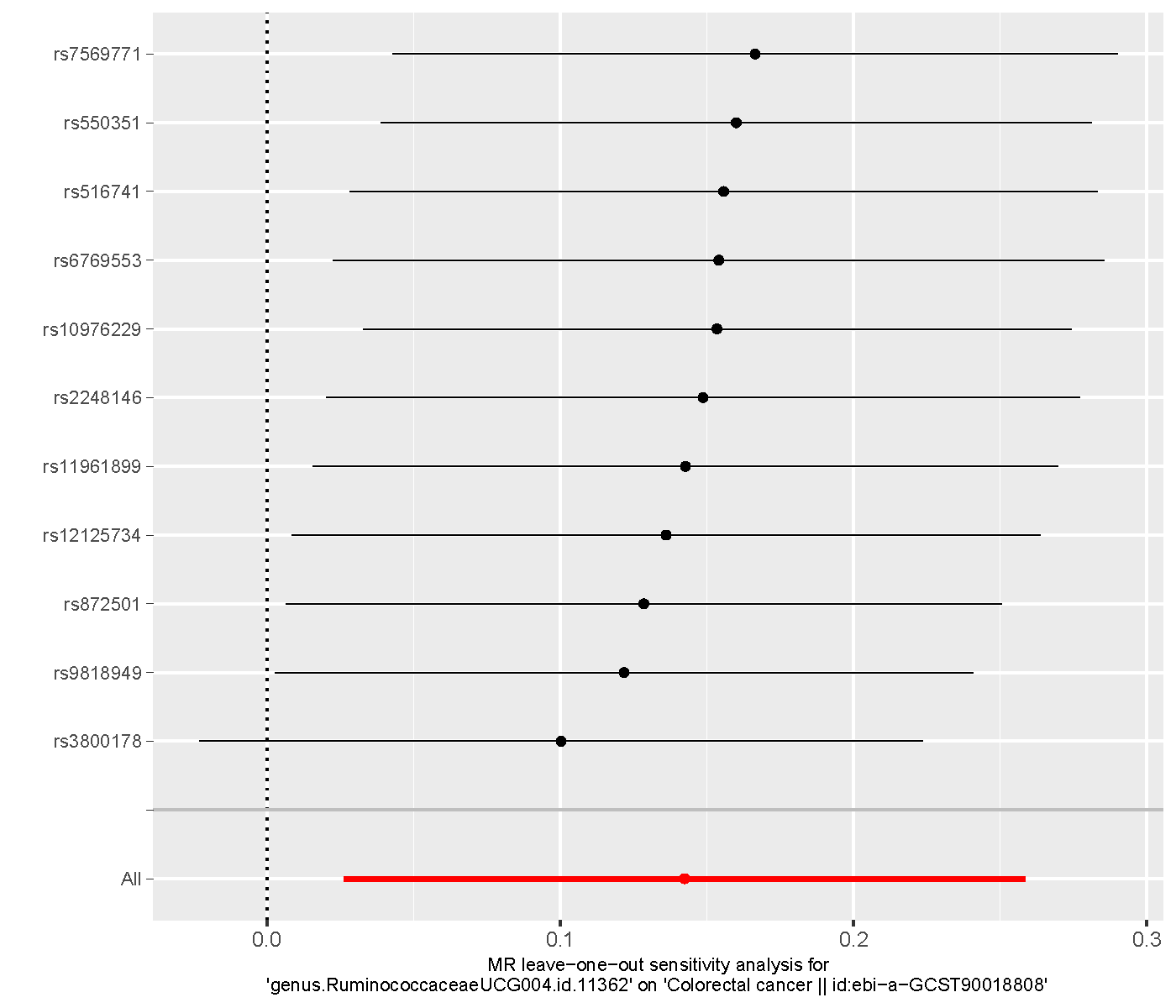

Supplement: Supplementary file 3 [file medi-105-e49652-s003.tiff]

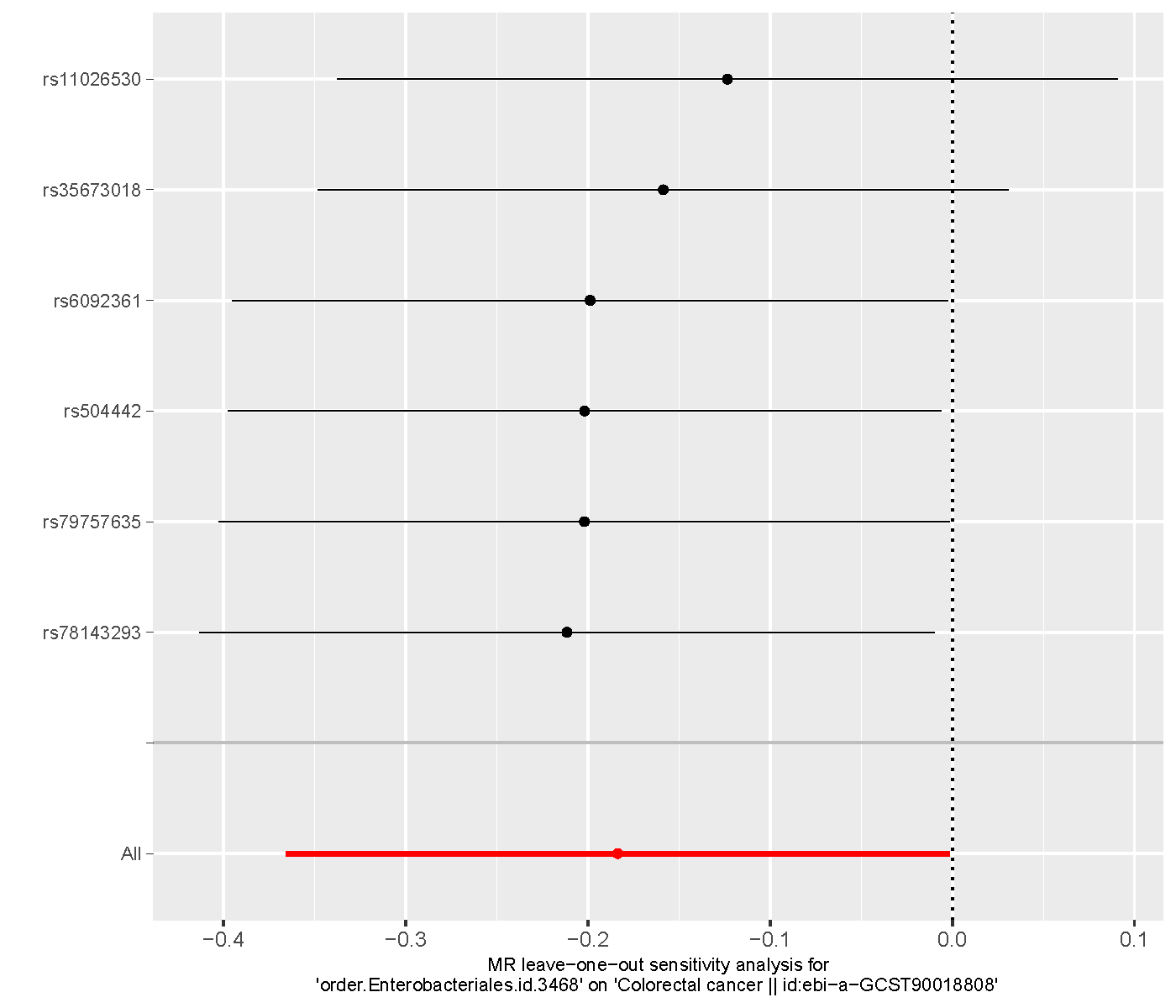

Supplement: Supplementary file 4 [file medi-105-e49652-s004.tiff]

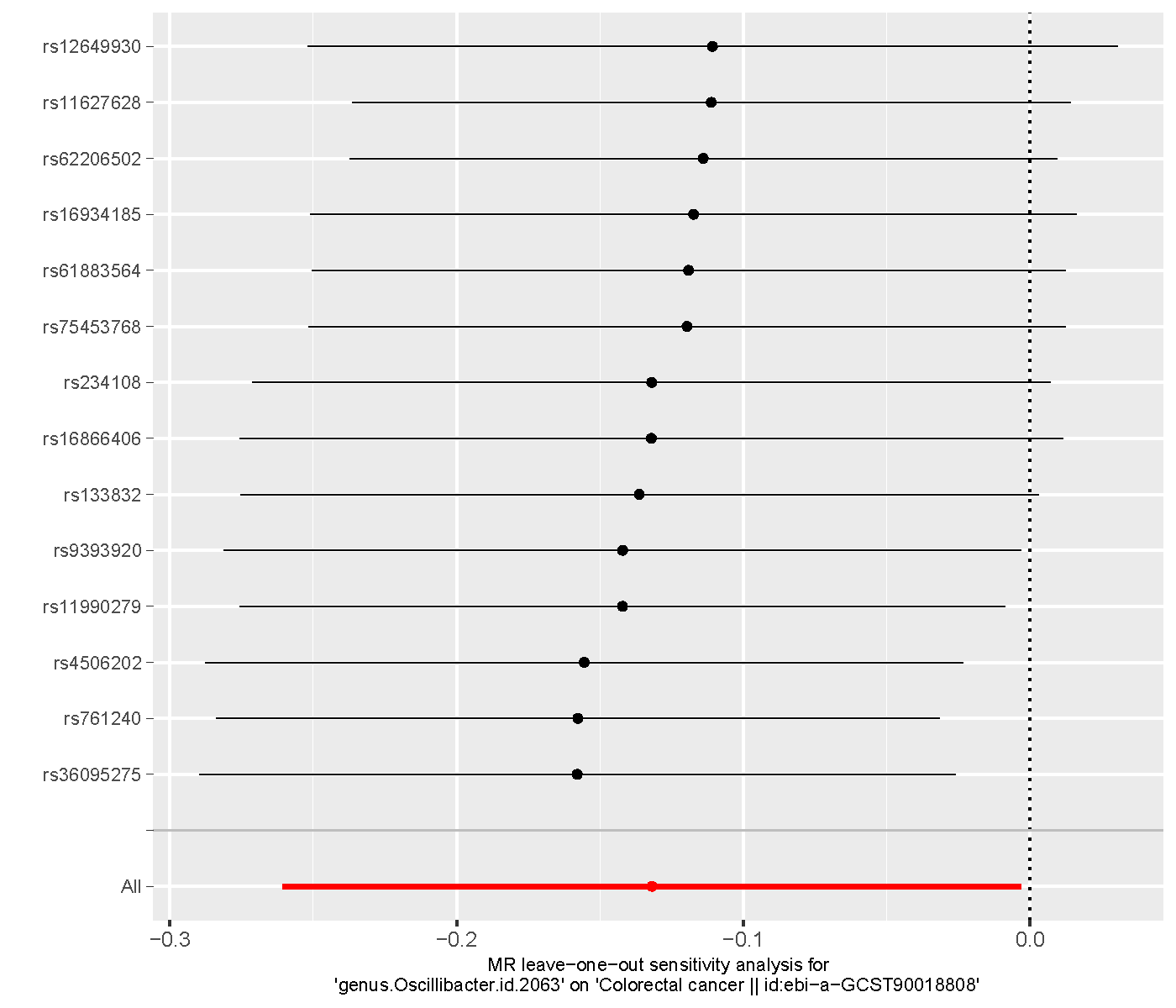

Supplement: Supplementary file 5 [file medi-105-e49652-s005.tiff]

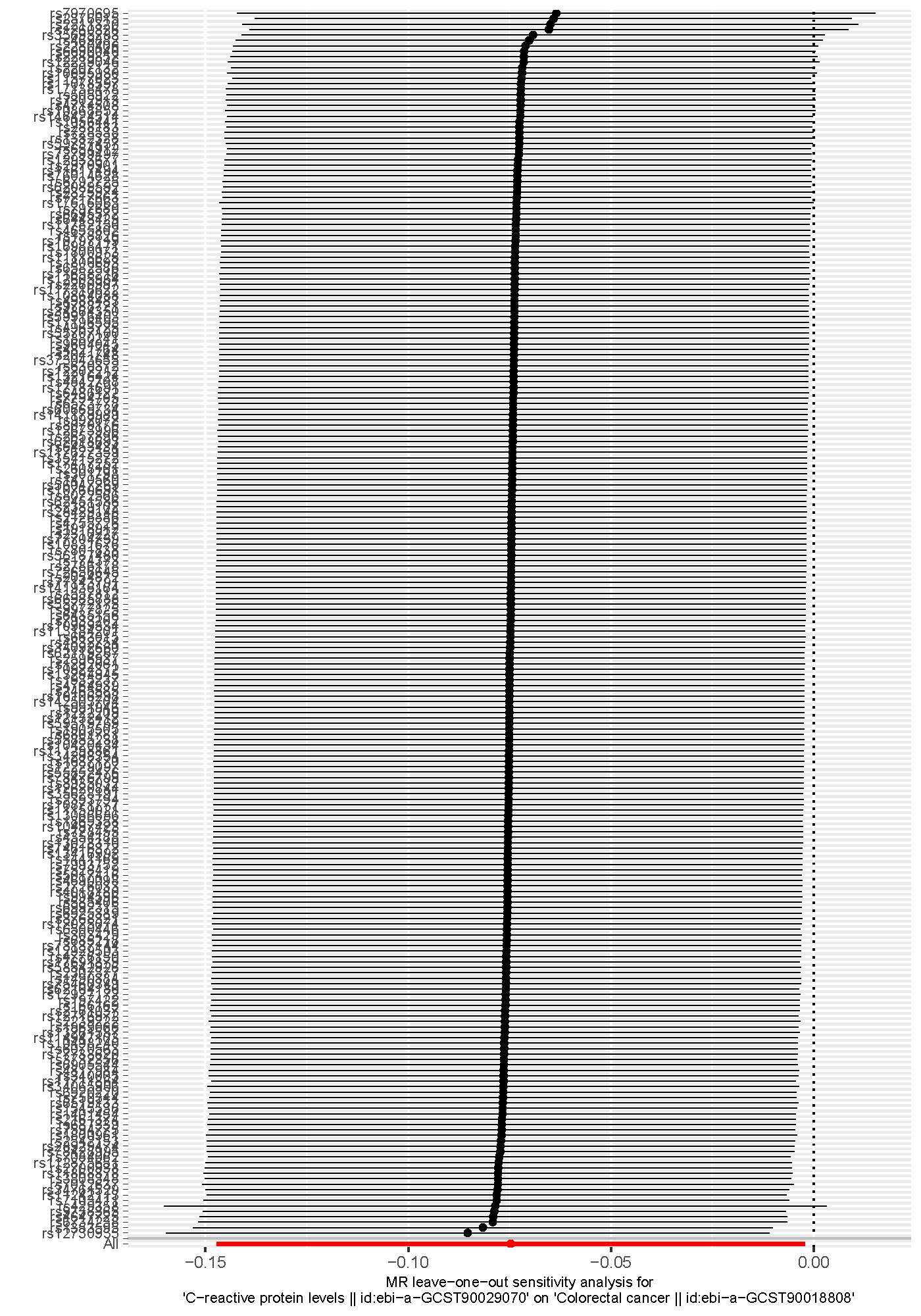

Supplement: Supplementary file 6 [file medi-105-e49652-s006.tif]
